# Supplementary material for: Enhancing violations of Leggett-Garg inequalities in nonequilibrium correlated many-body systems by interactions and decoherence
Source: Sci Rep. 2019 Nov 28;9:17772. doi: 10.1038/s41598-019-54121-1 (PMC6882789; doi:10.1038/s41598-019-54121-1)
Supplement: Supplementary file 1 — Supplementary information [file 41598_2019_54121_MOESM1_ESM.pdf]

— SUPPLEMENTARY INFORMATION —
Enhancing violations of Leggett-Garg inequalities in nonequilibrium correlated many-body systems by interactions and decoherence

J. J. Mendoza-Arenas[1], F. J. Gómez-Ruiz[2,1,*], F. J. Rodríguez[1], and L. Quiroga[1]

[1]Departamento de Física, Universidad de los Andes, A.A. 4976, Bogotá D. C., Colombia.
[2]Donostia International Physics Center, E-20018 San Sebastián, Spain.
[*]fj.gomez34@dipc.org

ABSTRACT

In this Supplementary Information (SI), we provide details of the analytical strategies employed in the main text to obtain exact numerical results for two-time correlations and Leggett-Garg inequalities for a nonequilibrium setup of quantum transport.

S1 Weakly-interacting system

In this section we briefly illustrate the impact of bulk dephasing on LGIs in the weakly-interacting regime ($\Delta < 1$) of the nonequilibrium model. In Fig. S1 we show how, similarly to spin transport, the value of the Leggett-Garg function for $\alpha = z$ monotonically decreases with the dephasing rate γ. Thus the quantumness of the transport supported by the system is degraded by environmental coupling, as intuitively expected[1]. It is worth noting that the inequalities are violated even for the largest values of dephasing considered, so the transport is of quantum nature and its properties cannot be accounted for classically.

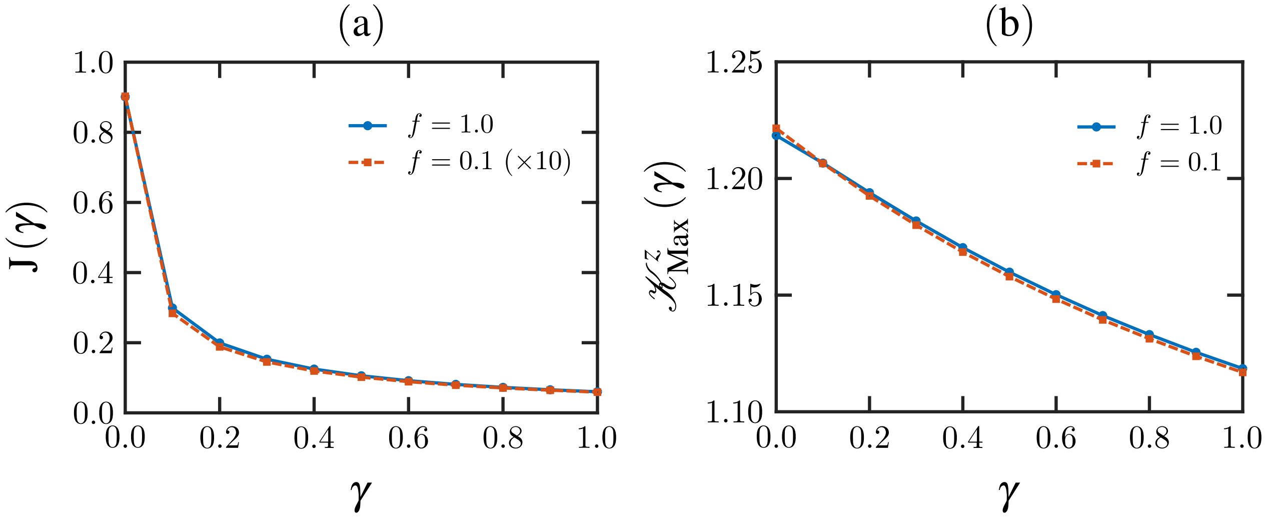

Figure S1. Weakly-interacting XXZ model. (a) Spin current as a function of dephasing, for $L = 60$ sites, $\Delta = 0.5$, $\alpha = z$ and both weak ($f = 0.1$) and strong ($f = 1.0$) driving. (b) Corresponding maximal value of Leggett-Garg functions.

S2 LGI violations for measurements on different sites

In this section we discuss how the main result of our work, namely the dephasing-enhanced LGI violation for strong interactions, is modified when we consider observables different to $\hat{\sigma}^z_{L/2}$. First we evaluate the two-time correlations for local operators

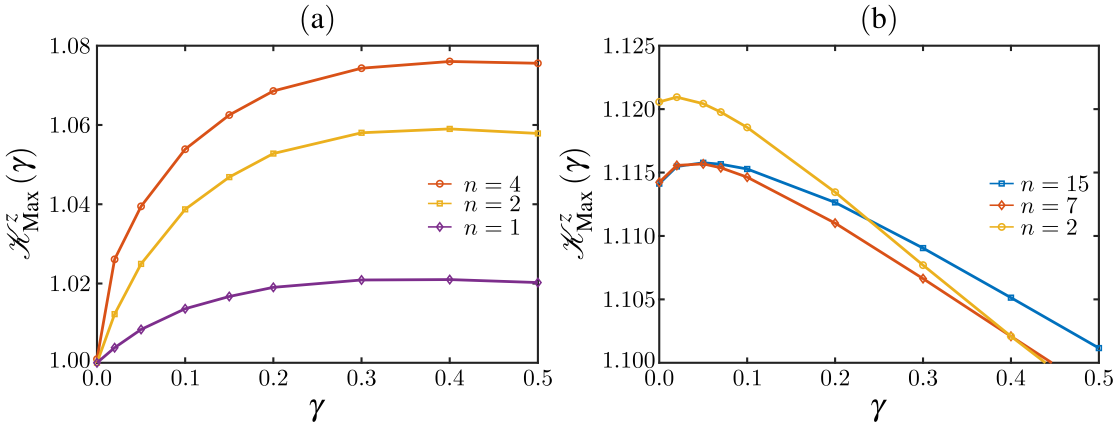

Figure S2. Dephasing-enhanced LGI violation for different sites n where correlations are measured. (a) At maximal driving $f = 1$ and $L = 16$. (b) At intermediate driving $f = 0.5$ and $L = 30$. We depict the maximal LGI violation for $\alpha = z$ as a function of dephasing rate γ, for $\Delta = 2$.

applied to different sites. The results are shown in Fig. S2. For maximal driving $f = 1$, the Leggett-Garg function has a very similar behavior to that evaluated at the central site (Fig. 4(a) of the main text), but with a lower violation since excitations created closer to the boundaries propagate more slowly. This is more clearly seen for weak dephasing, due to the presence of the ferromagnetic domains. In fact, for zero dephasing, no violation is seen for measurement sites away from the centre. For intermediate driving, deep inside the chain, the Leggett-Garg function is essentially site-independent for low dephasing rates γ; differences emerge for larger γ, for which the violation of LGIs starts to be degraded. For sites closer to the boundary the violation enhancement compared to $\gamma = 0$ is weaker, but still notable. Thus the main results of violation enhancement by dephasing are not exclusive to the central spin.

Now we consider the effect of non-local measurements. For this we take strings of operators $\hat{Q} = \hat{\sigma}^z_l \hat{\sigma}^z_{l+1} \hat{\sigma}^z_{l+2} \cdots \hat{\sigma}^z_n$ of different length, located around the central site of the lattice. The results, shown in Fig. S3, are qualitatively very similar to those obtained for the central site (and different sites, as seen in Fig. S2). For maximal driving we again see an enhancement of the violation even for large values of γ. However, as the string of $\hat{\sigma}^z_l$ operators includes sites closer to the boundaries, the maximal violation decreases; furthermore, for zero dephasing, no appreciable violation is seen for long-enough strings. For intermediate driving, the violation of the LGIs for several sites is also similar to that of a single site, with a very small but observable enhancement by dephasing. These results indicate that our observations remain qualitatively unaffected if non-local operators are considered instead of single-site operators.

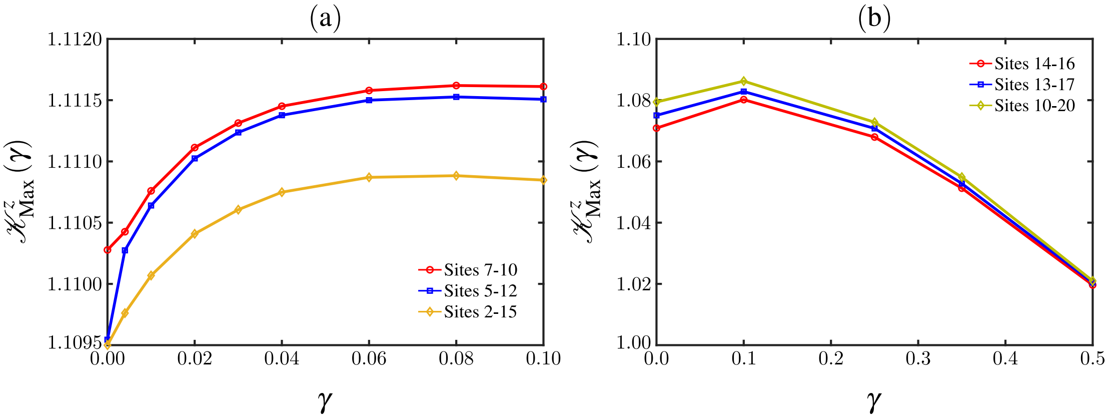

Figure S3. Dephasing-enhanced LGI violation for operators acting on several sites. (a) At maximal driving $f = 1$ and $L = 16$. (b) At intermediate driving $f = 0.5$ and $L = 30$. We depict the maximal LGI violation for $\alpha = z$ as a function of dephasing rate γ, for $\Delta = 2$.

S3 Analytic results on the minimal model

In the following we describe an analytic approach to obtain the Leggett-Garg functions of the simple minimal model, focused on large driving and weak dephasing. We first note that in the maximally-driven limit $f=1$ and in the absence of dephasing, this model features an insulating NESS akin to that of the original model of the main text, of the form[2]

$$\hat{\rho}^{(0)} = \frac{1}{\mathscr{N}}|\Psi\rangle\langle\Psi|, \quad |\Psi\rangle = \sum_{n=0}^{K-1}(2\delta)^{-n}|K-n\rangle, \tag{S1}$$

with normalization constant

$$\mathscr{N} = \frac{4\delta^2-(4\delta^2)^{1-K}}{4\delta^2-1}. \tag{S2}$$

This result shows that the population of a site exponentially decreases as it gets far from the rightmost site $|K\rangle$.

S3.1 Perturbative $1-f$ solution to the minimal model

Now we move from the maximally-driven case, and consider the situation where a weak back-flow is introduced, while keeping $\gamma=0$. We obtain the correction to the NESS to first order in $\mu=1-f\ll 1$, given by

$$\hat{\rho}_\infty = \hat{\rho}^{(0)}+\mu\hat{\rho}^{(1)}. \tag{S3}$$

For this we rewrite the Lindblad superoperator of Eq. (2) of the main text in the form

$$\hat{\mathscr{L}}(\hat{\rho}) = \hat{\mathscr{L}}^{(0)}(\hat{\rho})+\mu\hat{\mathscr{L}}^{(1)}(\hat{\rho}), \tag{S4}$$

where $\hat{\mathscr{L}}^{(0)}(\hat{\rho})$ corresponds to the $\mu=0$ Lindblad superoperator, namely

$$\hat{\mathscr{L}}^{(0)}(\hat{\rho}) = -\mathrm{i}\left[\hat{\mathscr{H}},\hat{\rho}\right]+\Gamma\left(\mathscr{D}_{\hat{\Lambda}_\ell^-}+\mathscr{D}_{\hat{\Lambda}_r^+}\right), \tag{S5}$$

and $\hat{\mathscr{L}}^{(1)}(\hat{\rho})$ to the remaining terms,

$$\hat{\mathscr{L}}^{(1)}(\hat{\rho}) = \frac{\Gamma}{2}\left(\mathscr{D}_{\hat{\Lambda}_\ell^+}+\mathscr{D}_{\hat{\Lambda}_r^-}-\mathscr{D}_{\hat{\Lambda}_\ell^-}-\mathscr{D}_{\hat{\Lambda}_r^+}\right), \tag{S6}$$

where each dissipator $\mathscr{D}$ is given by

$$\mathscr{D}_{\hat{X}}(\hat{\rho}) = \hat{X}\hat{\rho}\hat{X}^\dagger-\frac{1}{2}\{\hat{X}^\dagger\hat{X},\hat{\rho}\}. \tag{S7}$$

To solve Eq. (S4) for the NESS $\hat{\rho}_\infty$, we use the anstaz

$$\hat{\rho}_\infty = \sum_{p=0}^{\infty}\mu^p\hat{\rho}^{(p)}. \tag{S8}$$

Gathering terms of equal powers of μ, we obtain the equations that determine the different order corrections. For $p=0$, this corresponds to $\hat{\mathscr{L}}^{(0)}\left(\hat{\rho}^{(0)}\right)=0$, which is the $\mu=0$ equation with the solution of Eq. (S1). For $p>0$ we have

$$\hat{\mathscr{L}}^{(0)}(\hat{\rho}^{(p)})+\hat{\mathscr{L}}^{(1)}(\hat{\rho}^{(p-1)})=0, \tag{S9}$$

which indicates that to obtain the correction of $\mathscr{O}(p)$, that of $\mathscr{O}(p-1)$ is required. Importantly, since $\hat{\rho}^{(0)}$ is a valid density matrix, its trace is one, so the $p>0$ corrections are traceless. In the following calculation we restrict to $p=1$, valid for a weak deviation from the maximally-driven system. Equation (S9) becomes

$$\hat{\mathscr{L}}^{(0)}(\hat{\rho}^{(1)})+\hat{\mathscr{L}}^{(1)}(\hat{\rho}^{(0)})=0, \tag{S10}$$

from which we can obtain $\hat{\rho}^{(1)}$ from $\hat{\rho}^{(0)}$. First we calculate $\hat{\mathscr{L}}^{(1)}(\hat{\rho}^{(0)})$, obtaining

$$\hat{\mathscr{L}}^{(1)}(\hat{\rho}^{(0)}) = \frac{\Gamma}{2\mathscr{N}}\left\{|s\rangle\langle s|\left(1+(2\delta)^{-2(K-1)}\right)-\frac{1}{2}\sum_{n=0}^{K-1}(2\delta)^{-n}(|K\rangle\langle K-n|+\text{H.c.})-\frac{1}{2}\sum_{n=0}^{K-1}(2\delta)^{-(K-1+n)}(|1\rangle\langle K-n|+\text{H.c.})\right\}.$$

$$ \tag{S11} $$

To calculate $\hat{\mathscr{L}}^{(0)}(\hat{\rho}^{(1)})$ we consider the following general form for $\hat{\rho}^{(1)}$,

$$\hat{\rho}^{(1)} = a_s|s\rangle + \sum_{m,n=0}^{K-1} a_{m,n}|K-m\rangle\langle K-n|. \tag{S12}$$

Note that site $|s\rangle$ has no coherences with other sites, as it is only incoherently coupled to other sites ($|1\rangle$ and $|K\rangle$). We obtain

$$\begin{aligned}\hat{\mathscr{L}}^{(0)}(\hat{\rho}^{(1)}) &= \Gamma a_s|K\rangle\langle K| + \Gamma(a_{K-1,K-1}-a_s)|s\rangle\langle s| - \frac{\Gamma}{2}\sum_{m=0}^{K-1}(a_{K-1,m}|1\rangle\langle K-m| + \text{H.c.})\\ &+ \frac{1}{2}\sum_{m=0}^{K-1}\sum_{n=1}^{K-1}(ia_{m,n}|K-m\rangle\langle K-n+1| + ia_{m,n-1}|K-m\rangle\langle K-n|) + \text{H.c.}) + \delta\sum_{m=1}^{K-1}(ia_{n,0}|K-n\rangle\langle K| + \text{H.c.}).\end{aligned} \tag{S13}$$

After gathering the coefficients $a_{m,n}$ of all the different basis elements $|K-m\rangle\langle K-n|$ in Eqs. (S11) and (S13), and considering that $a^*_{m,n} = a_{n,m}$ so the density matrix is Hermitian, we obtain a linear system of equations whose solution gives the correction $\hat{\rho}^{(1)}$. Rewriting each correction coefficient as $a_{m,n} = r_{m,n} + i\,j_{m,n}$ and considering the Hermiticity of the density matrix ($r_{m,n} = r_{n,m}$ and $j_{m,n} = -j_{n,m}$), this is transformed into a linear system of $1+K^2$ equations with $(K^2+K+2)/2$ unknowns $r_{m,n}$ (including $r_s \equiv a_s$) and $(K^2-K)/2$ unknowns $j_{m,n}$ (the diagonal elements must be real). In addition, since $\hat{\rho}^{(1)}$ is traceless, the diagonal coefficients must also satisfy

$$r_s + \sum_{n=0}^{K-1} r_{n,n} = 0. \tag{S14}$$

We solve this system of equations up to $\mathscr{O}(\delta^{-2})$, for which $\hat{\rho}^{(1)}$ has the (almost tridiagonal) form

$$\hat{\rho}^{(1)} = \begin{pmatrix} r_{K-1} & -i\,j_1 & & & \cdots & & & & & \\ i\,j_1 & r_b & -i\,j_1 & & \cdots & & & & & \\ & i\,j_1 & r_b & -i\,j_1 & \cdots & & & & & \\ & & & & \ddots & & & & & \\ & & & & \cdots & i\,j_1 & r_b & -i\,j_1 & r_{0,2} & \\ & & & & \cdots & & i\,j_1 & r_1 & r_{0,1}-i\,j_1 & \\ & & & & \cdots & & r_{0,2} & r_{0,1}+i\,j_1 & r_0 & \\ & & & & \cdots & & & & & r_s \end{pmatrix} \tag{S15}$$

The lattice populations have four possible values, corresponding to boundary (r_{K-1}, r_1, r_0) and bulk (r_b) values, given by

$$\begin{aligned} r_{K-1} &= \frac{1}{2}\left(\frac{1}{2\delta}\right)^2, \qquad r_b = (1+\Gamma^2)r_{K-1},\\ r_1 &= \Gamma^2 r_{K-1}, \qquad r_0 = -r_{K-1}(1+\Gamma^2)(K-2) - \frac{1}{2}. \end{aligned} \tag{S16}$$

The off-diagonal real values are

$$r_{0,1} = -\frac{1}{4\delta}, \qquad r_{0,2} = -\frac{1}{2}\left(\frac{1}{2\delta}\right)^2. \tag{S17}$$

The imaginary values correspond to the expectation value of the (homogeneous) NESS current, given by

$$j_1 = \frac{\langle \hat{\mathrm{J}}_{\min,k}\rangle}{\mu} = \frac{1}{2}\Gamma\left(\frac{1}{2\delta}\right)^2. \tag{S18}$$

Finally, the population of the auxiliary state is $r_s = 1/2$. We have verified numerically that these results indeed correspond to the correct solution to the linear system of equations, and thus give the correct form of the first-order correction $\hat{\rho}^{(1)}$.

S3.2 LGIs violation enhancement by driving

Here we discuss the Leggett-Garg functions of the NESS of the strongly-driven minimal model. We first consider the $f = 1$ limit. Applying the operator $\hat{Q} = \hat{\mathscr{I}} - 2|P\rangle\langle P|$ to $\hat{\rho}^{(0)}$ we get

$$\hat{Q}\hat{\rho}^{(0)} = \hat{\rho}^{(0)} - \frac{2}{\mathscr{N}} \sum_{n=0}^{K-1} (2\delta)^{-(K-P+n)} |P\rangle\langle K-n|. \tag{S19}$$

The dominant term of the sum is of $\mathscr{O}((2\delta)^{-(K-p)})$ (for $n = 0$), which is insignificant within the order $O(\delta^{-2})$ of our solution if site P is far from the right boundary. Thus $\hat{Q}\hat{\rho}^{(0)} \approx \hat{\rho}^{(0)}$, and the time correlation is

$$\mathscr{C}^{(0)}(t) = \mathrm{Tr}\left(\hat{\mathrm{Q}}\exp[\hat{\mathscr{L}}^{(0)}\mathrm{t}]\hat{\mathrm{Q}}\hat{\rho}^{(0)}\right) \approx \mathrm{Tr}\left(\hat{\mathrm{Q}}\exp[\hat{\mathscr{L}}^{(0)}\mathrm{t}]\hat{\rho}^{(0)}\right) = \mathrm{Tr}\left(\hat{\mathrm{Q}}\hat{\rho}^{(0)}\right) \approx \mathrm{Tr}(\hat{\rho}^{(0)}) = 1. \tag{S20}$$

It is approximately constant, as expected from the insulating nature of the state. The corresponding Leggett-Garg function is

$$\mathscr{K}^{(0)}(t) = 2\mathscr{C}^{(0)}(t) - \mathscr{C}^{(0)}(2t) \approx 1, \tag{S21}$$

indicating that the LGI is not violated[1].

Now we consider the system slightly below maximal driving, namely with $\mu = 1 - f \ll 1$, whose NESS was discussed in Section S3.1. With the NESS of Eq. (S3) calculated up to first order in μ we can proceed to obtain the perturbative correction to the early-time time correlations $\mathscr{C}^{(1)}(t)$ and Leggett-Garg function $\mathscr{K}^{(1)}(t)$, so that

$$\mathscr{C}(t) = \mathscr{C}^{(0)}(t) + \mu\mathscr{C}^{(1)}(t), \tag{S22}$$

$$\mathscr{K}(t) = \mathscr{K}^{(0)}(t) + \mu\mathscr{K}^{(1)}(t). \tag{S23}$$

Expanding the time correlations in Eq. (3) of the main text up to second order in time, the perturbative correction to the $f = 1$ Leggett-Garg function is

$$\mathscr{K}^{(1)}(t) = -t^2 \mathrm{Re}\left(\mathrm{Tr}\left[\hat{\mathrm{Q}}\hat{\mathscr{L}}^2\hat{\mathrm{Q}}\hat{\rho}^{(1)}\right]\right), \tag{S24}$$

indicating that there is no linear correction in time for $\mathscr{K}$. To calculate it, first we note that applying $\hat{Q}$ to $\hat{\rho}^{(1)}$ gives

$$\hat{Q}\hat{\rho}^{(1)} = \hat{\rho}^{(1)} - 2r_b|P\rangle\langle P| + 2ij_1\left(|P\rangle\langle P+1| - |P\rangle\langle P-1|\right). \tag{S25}$$

Applying the Lindblad operator $\hat{\mathscr{L}}$ twice to $\hat{Q}\hat{\rho}^{(1)}$ is a lengthy process, so we focused on obtaining the real diagonal terms which give a finite contribution to the trace after being multiplied again by $\hat{Q}$. We finally find that

$$\mathrm{Re}\left(\mathrm{Tr}\left[\hat{\mathrm{Q}}\hat{\mathscr{L}}^2\hat{\mathrm{Q}}\hat{\rho}^{(1)}\right]\right) = -2\mathrm{Re}\left(\mathrm{Tr}|\mathrm{P}\rangle\langle\mathrm{P}|2\mathrm{r_b}|\mathrm{P}\rangle\langle\mathrm{P}|\right) = -4r_b, \tag{S26}$$

which, using Eq. (S16), gives that the correction of the LGI function is

$$\mathscr{K}(t) - \mathscr{K}^{(0)}(t) = 2(1-f)(1+\Gamma^2)\left(\frac{t}{2\delta}\right)^2. \tag{S27}$$

Since this is a positive quantity and $\mathscr{K}^{(0)}(t) \approx 1$, we indeed observe that LGIs are violated when moving slightly away from the $f = 1$ scenario, and that such violation increases with time and as f decreases. In Fig. S4(a) we show that this prediction agrees well with exact numerical results for early times.

S3.3 LGIs violation enhancement by dephasing

An identical calculation can be performed for the maximally-driven $f = 1$ limit, but with a weak dephasing rate $\gamma \ll 1$. However this can also be obtained considering the driving-dephasing equivalence in the minimal model described in Ref.[2], both were seen to induce the same effect on the NESS when replacing

$$\gamma = \frac{1}{2}(1-f)\frac{\Gamma}{4}. \tag{S28}$$

[1] We have observed numerically that $\mathscr{K}^{(0)}(t)$ is actually slightly larger than one for short times, but since the governing term is of $\mathscr{O}((2\delta)^{-(K-P)})$, such a violation is insignificant for $\delta > 1$.

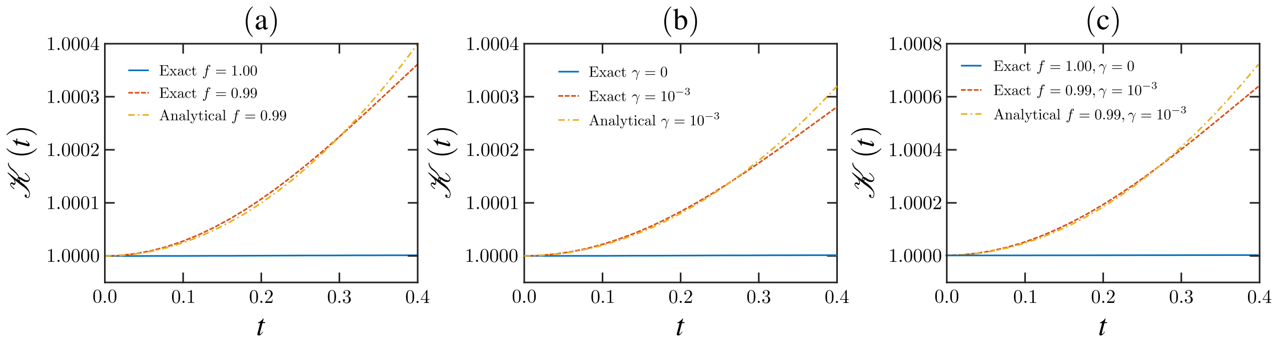

Figure S4. Comparison of analytical and numerical results for the minimal model. The results correspond to $K = 10$, $\delta = 2$ and $\Gamma = 1$, (a) for $f = 0.99$ and $\gamma = 0$, (b) for $f = 1.00$ and $\gamma = 10^{-3}$, (c) for $f = 0.99$ and $\gamma = 10^{-3}$.

The correction to the Leggett-Garg function is then

$$\mathscr{K}(t) - \mathscr{K}^{(0)}(t) = 16\gamma \frac{(1+\Gamma^2)}{\Gamma} \left(\frac{t}{2\delta}\right)^2, \tag{S29}$$

which is also positive and thus indicates that dephasing induces a violation of the LGIs, which increases with γ. This result is also in agreement with exact numerical calculations, as depicted in In Fig. S4(b). If both a weak backflow and dephasing are present, where both mechanisms enhance the LGI violations, the result is simply the sum of the independent contributions of Eqs. (S27) and (S29), namely

$$\mathscr{K}(t) - \mathscr{K}^{(0)}(t) = 2(1+\Gamma^2)\left[(1-f) + \frac{8\gamma}{\Gamma}\right]\left(\frac{t}{2\delta}\right)^2. \tag{S30}$$

This additive enhancement of the two mechanisms is verified by exact numerical simulations, which as shown in Fig. S4(c), present a very good agreement.

References

1. Lambert, N., Emary, C., Chen, Y.-N. & Nori, F. Distinguishing Quantum and Classical Transport through Nanostructures. *Phys. Rev. Lett.* **105**, 176801, DOI: 10.1103/PhysRevLett.105.176801 (2010).
2. Mendoza-Arenas, J. J., Grujic, T., Jaksch, D. & Clark, S. R. Dephasing enhanced transport in nonequilibrium strongly correlated quantum systems. *Phys. Rev. B* **87**, 235130, DOI: 10.1103/PhysRevB.87.235130 (2013).
